# Type 2 Innate Lymphocytes Actuate Immunity Against Tumours and Limit Cancer Metastasis

**DOI:** 10.1038/s41598-018-20608-6

**Published:** 2018-02-13

**Authors:** Iryna Saranchova, Jeffrey Han, Rysa Zaman, Hitesh Arora, Hui Huang, Franz Fenninger, Kyung Bok Choi, Lonna Munro, Cheryl G. Pfeifer, Ian Welch, Fumio Takei, Wilfred A. Jefferies

**Affiliations:** 1 The Michael Smith Laboratories, University of British Columbia, 301-2185 East Mall, Vancouver, V6T 1Z4 BC Canada; 2 The Vancouver Prostate Centre, Vancouver Coastal Health Research Institute, Vancouver, Canada; 3 Department of Microbiology & Immunology, University of British Columbia, Vancouver, Canada; 4 Department of Animal Care Services, University of British Columbia, Vancouver, Canada; 5 Terry Fox Laboratory, BC Cancer Agency, Vancouver, Canada; 6 Department of Pathology & Laboratory Medicine, University of British Columbia, Vancouver, Canada; 7 Department of Medical Genetics, University of British Columbia, Vancouver, Canada; 8 Department of Zoology, University of British Columbia, Vancouver, Canada; 9 The Centre for Blood Research, University of British Columbia, Vancouver, Canada; 10 Djavad Mowafaghian Centre for Brain Health, University of British Columbia, Vancouver, Canada

## Abstract

Type 2 innate lymphoid cells (ILC2) potentiate immune responses, however, their role in mediating adaptive immunity in cancer has not been assessed. Here, we report that mice genetically lacking ILC2s have significantly increased tumour growth rates and conspicuously higher frequency of circulating tumour cells (CTCs) and resulting metastasis to distal organs. Our data support the model that IL-33 dependent tumour-infiltrating ILC2s are mobilized from the lungs and other tissues through chemoattraction to enter tumours, and subsequently mediate tumour immune-surveillance by cooperating with dendritic cells to promote adaptive cytolytic T cell responses. We conclude that ILC2s play a fundamental, yet hitherto undescribed role in enhancing anti-cancer immunity and controlling tumour metastasis.

## Introduction

Our understanding of the role of the immune response in suppressing the emergence of malignancies and limiting metastases continues to evolve. It has been generally accepted that the local microenvironment influences the regulatory processes in tumour tissue via chemokine- and cytokine-related signalling pathways, highlighting context-specific biological functions of the tumour tissue framework. One of the prompt responders to tissue insult is a collection of Innate Lymphoid Cells (ILCs), which can modify immune responses to the needs of local tissue microenvironment. ILCs are currently divided into three main groups, which are defined by cell-surface markers and by the cytokines they produce^1^. Two ILC subsets have been implicated in tumour immunity, including Group 1 ILCs (ILC1 or natural killer (NK) cells)^2^ and Group 3 ILCs (ILC3)^3–5^. However, the role of Group 2 ILCs (ILC2) in tumour immune-surveillance has not been determined.

ILC2 cells lack adaptive antigen receptors, sense the microenvironment via cytokine receptors, and regulate the developing immune response via further secretion of a wide array of specific cytokines^6–12^ and through the expression of major histocompatibility class II (MHC-II) molecules^13^. Secretion of IL-13 by ILC2s is important for the migration of activated dendritic cells (DCs) to the draining lymph nodes^8^, where T cell priming and activation takes place. Additionally, IL-13 secretion by ILC2s at early stages of tumour development can drive the production of eosinophil chemo-attractant, eotaxin, by epithelial cells^14,15^ with consequent eosinophil recruitment^9,16^. Upon arrival at the site of an immunological response, eosinophils require IL-5 for activation and survival^17^, which is also secreted by ILC2s^11^. It is reported that eosinophils may promote tumour rejection through secretion of CD8^+^ and CD4^+^ T cell chemo-attractants, such as CXCL9, CXCL10, CCL5 (via STAT1) or CCL17, CCL22 (via STAT6), which allow the trafficking of T cells to the tumour site^18^. Moreover, ILC2s are capable of influencing adaptive immune responses through cell-to-cell contact via MHC-II molecules that they express on their cell surface^6,13^. Finally, for proper ILC2 development and function, IL-33 is required in the microenvironment^11,19,20^.

We previously demonstrated that IL-33 expression is reduced in clinical specimens from patients with prostate and renal carcinomas upon their transition from a primary to a metastatic form^21^. We also demonstrated that re-introducing IL-33 into metastatic murine tumours increases expression of antigen processing components including TAP-1 and MHC-I surface expression and augments cytotoxic T cell (CTL) immune recognition^21^. Moreover, down-modulation of IL-33, together with down-modulation of antigen processing machinery and MHC-I-related genes during the primary to metastatic transition in tumours, represents a newly defined form of tumour immune-escape. Based on these clues, we hypothesized that since ILC2s are developmentally and functionally dependent on IL-33, ILC2s may have an undescribed role in promoting and mediating immune responses against tumours. As a test of this hypothesis, we examine whether the lack of ILC2s supports tumour progression. These data help to revise our knowledge of immunity to emerging and metastatic malignancies.

## Results

### Tumour study models

Currently, the tumour mutational landscape and eventual treatment decision commonly rely on the molecular profiling of the primary tumour at early stages, without information on possible genetic and epigenetic alteration during disease progression and metastasis. Thus, gene expression profiling of primary tumours and assessing mutational changes accumulated over time in antecedent metastatic lesions and/or local recurrences may help to elucidate the mechanism of transition from primary tumour to its metastatic form, increase therapeutic success and lead to a reduction of systemic relapse of the disease.

**Figure 1:**
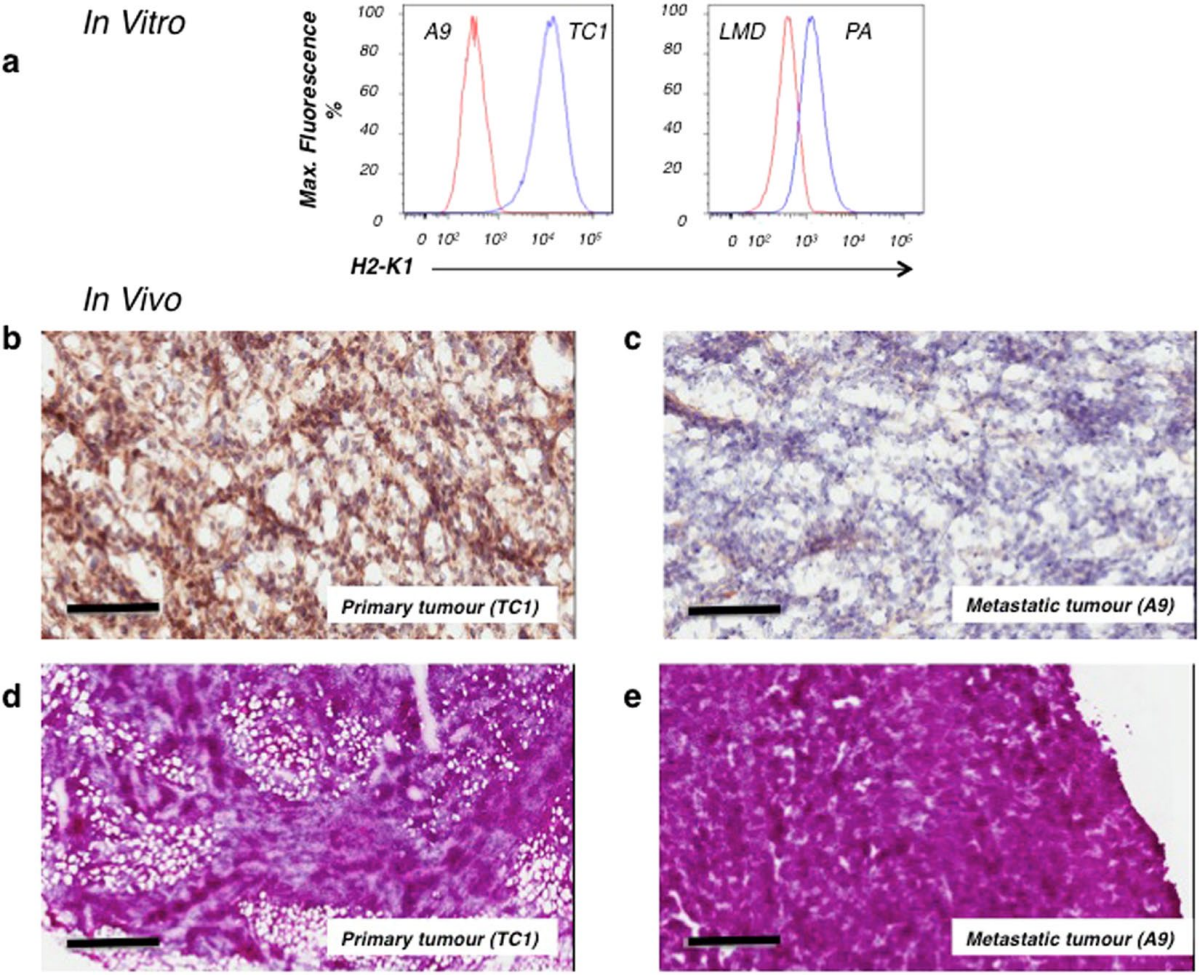
The study models of antecedent tumour cell lines, TC1/A9, retain their immunological characteristics both *in vitro* and *in vivo*. (**a**) Flow cytometry shows the MHC-I expression levels of tumour cells grown *in vitro*, where the primary cells (TC1) express higher levels of MHC-I than their metastatic derivatives (A9). (**b**,**c**) Resected tumour sections were stained with antibodies against MHC-I. (**b**) Primary TC1 tumours retain MHC-I expression *in vivo*. (**c**) The corresponding metastatic A9 tumour has greatly reduced MHC-I expression. (**d**,**e**) Morphology of tumours is shown with H&E staining. (**d**) Primary TC1 tumours resemble a sponge structure that clearly distinguishes them from (**e**) metastatic A9 tumours, which appear uniformly distributed, with a solid architecture. 10 µm thick sections were imaged at 20x (**b**,**c**) or 4x (**d**,**e**) magnification. Size bar = 100 µm in (**b**,**c**); Size bar = 500 µm in (**d**,**e**).

In this study, we utilized a matched pair of antecedent murine primary and metastatic tumour lines. We have selected a previously published murine tumour model, which represents both primary tumour cells and metastatic cells arising from an initial primary lung tumour: primary TC1 tumours and metastatic A9 tumours^22^. The primary TC1 tumour is a murine lung primary tumour model that we have previously shown to produce IL-33 and to express MHC-I on its surface^21,22^. The metastatic murine lung tumour (A9) spontaneously arose from the primary tumour cells (TC1) during a tumour immunization challenge in a mouse. We have previously shown the metastatic A9 tumours to exhibit a great reduction in IL-33 production^21,22^ and to be MHC-I and antigen processing-deficient^23^ (Fig. 1a). The transition to an immunosubversive phenotype correlates with tumour aggressiveness and metastatic potential^22^. The matched pair of primary and metastatic cell lines were selected for three reasons: they reflect the different immunological properties of tumours (immune responsiveness versus immune evasiveness/escape); they retain these properties upon transplantation *in vivo* (Fig. 1b,c); and as an antecedent tumour pair, they allow for the detection of specific genetic and epigenetic changes acquired during primary tumour reprogramming toward its metastatic form.

A comparative morphological study of the tumours grown in mice from antecedent cell lines, TC1 and A9, revealed specific differences in tissue architecture and formation of tumour margins, consistent for primary and metastatic tumours. The primary TC1 tumour resembled a sponge structure (Fig. 1d) that appeared to be surrounded by a fibrous capsule. The metastatic A9 tumours appeared as dense sheets of cells (Fig. 1e), with no indication of a surrounding capsule and therefore, more able to disseminate to distal organs. We further studied the metastatic spread of TC1 and A9 tumour cells *in vivo*, as assessed by the appearance of EGFP-positive circulating tumour cells (CTCs) beyond the initial site of tumour inoculation (Table 1). CTCs arising from the subcutaneously implanted (s.c.) metastatic A9 tumours were detected in distal organs (*e.g*. adrenal glands, lung, brain), whereas CTCs from s.c. TC1 tumours were not detected in any of the tested tissues and organs of animals bearing primary tumours. The TC1 tumours did not appear to be genetically programmed to disseminate to distal sites (Table 1). Thus, our *in vitro* and *in vivo* studies demonstrate the reliability of matched pairs of antecedent tumour lines to study the transition of primary tumour to its metastatic form.

**Table 1:** Circulating tumour cells originating from metastatic tumours can be detected in distal organs. Primary TC1 and metastatic A9 tumour cells stably transfected with an EGFP-expressing vector were used to established subcutaneous tumours in WT C57Bl/6 mice. The appearance of EGFP-positive circulating tumour cells beyond the original site in distal organs was detected through the use of flow cytometry.

Tumour cells	Liver	Adrenal glands	Lungs	Blood	Brain
Metastatic A9	24.0–32.3%	8.7–14.9%	0.0–1.3%	Single cells (1–33 cells per o.5 ml)	0.0%
Primary TC1	0.0%	0.0%	0.0%	0.0%	0.0%

CTCs originating from metastatic tumours can be detected in distal organs.

### Tumour-infiltrating immune cells

**Figure 2:**
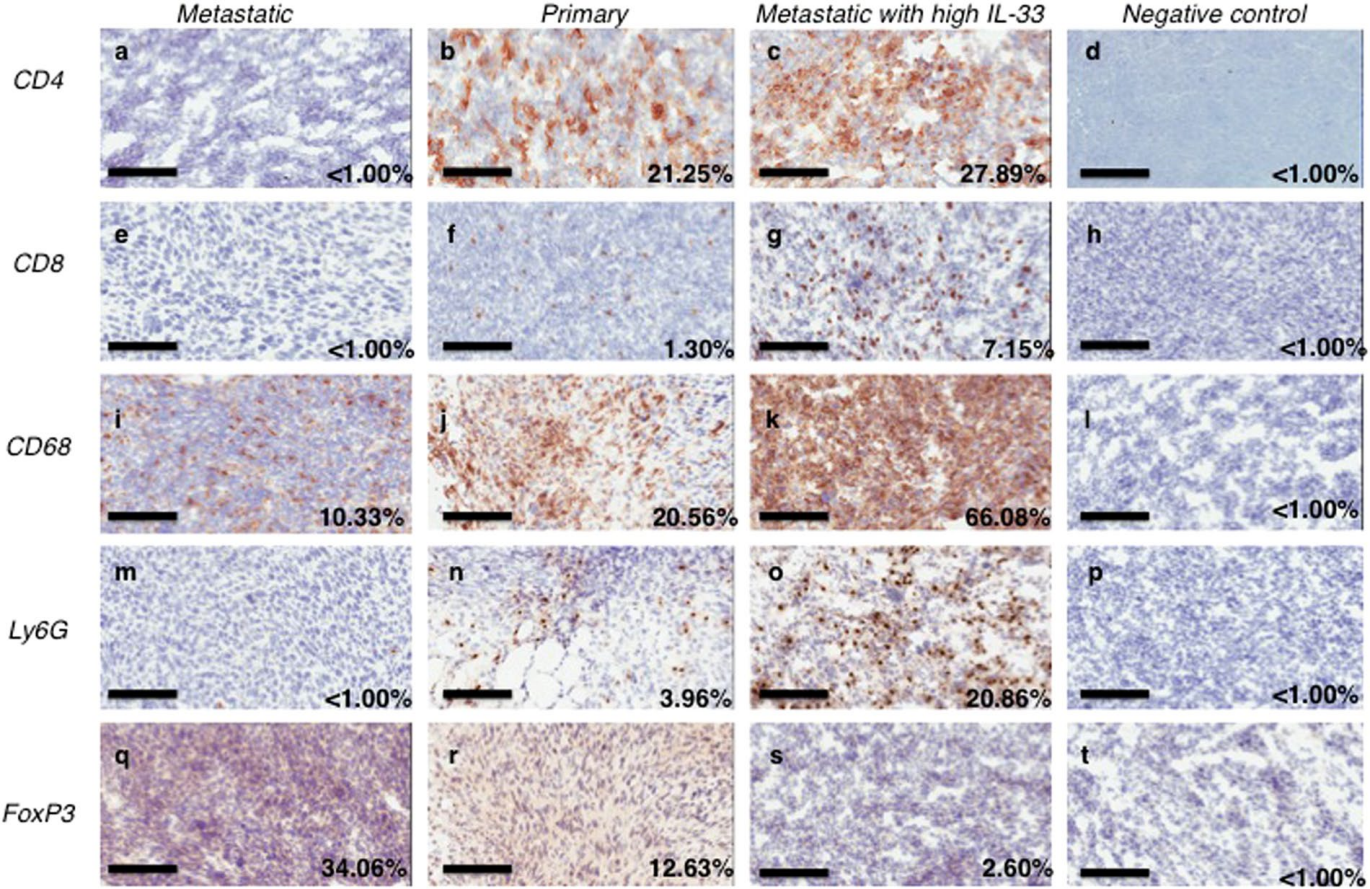
Genetic complementation of immune evasive tumours shows a clear phenotypic shift towards immune recognition. Immunohistochemical staining was used to visualise tumour infiltrating immune cells. CD4 is a glycoprotein found on the surface of T helper cells, macrophages, dendritic cells. CD8 is primarily a marker for cytotoxic T cells, but also found on natural killer cells and DCs; CD68 is a marker of monocytes and macrophages; Ly6G is a marker for neutrophils; FoxP3 is a marker of regulatory T cells. Greater numbers of CD4^+^ and CD8^+^ cells can be seen within (**c**,**g**) the genetically modified (metastatic A9+IL-33) tumours versus unmodified metastatic (A9) tumours (**a**,**e**). Fewer regulatory T cells are present in IL-33 expressing tumours, as indicated by lower FoxP3 staining; metastatic A9 (**q**) versus metastatic A9+IL-33 (**s**) or primary TC1 (**r**) tumours. Increased macrophage and neutrophil responses are seen in IL-33 expressing tumours: unmodified metastatic A9 (**i**,**m**) versus metastatic A9+IL-33 (**k**,**o**) or primary TC1 (**j**,**n**) tumours respectively. (**d**,**h**,**l**,**p**,**t**) Negative controls (rat IgG targeting Keyhole Limpet Hemocyanin (KLH)) were included to show that non-specific staining was minimized. The percentage of positively stained cells was calculated from the total number of cells on each slide. 10 µm thick sections were stained with appropriate antibodies and imaged at 20X magnification (**a**–**t**); size bar = 100 µm (**a**–**t**).

Tumour-infiltrating immune cells affect the kinetics of tumour growth^24,25^. The involvement of innate and adaptive immunity into tumour progression was assessed by immunohistochemical visualization of tumour tissue sections. The data demonstrates increased numbers of CD4^+^ and CD8^+^ TILs, macrophages and neutrophils, within primary tumours or metastatic IL-33-expressing tumours (Fig. 2b,c,f,g,j,k,n,o) when compared to metastatic tumours alone (Fig. 2a,e,i,m). The metastatic tumour microenvironment contains increased numbers of suppressive T-regulatory cell markers (FoxP3^+^) that are also associated with tumour immune-resistance (Fig. 2q,r,s,t). Thus the expression of IL-33 by the tumours diminished the accumulation of immune suppressive cells and promoted elevated numbers of infiltrating CD68^+^ tumour-associated macrophages (Fig. 2i,j,k,l) and neutrophils **(**Fig. 2m,n,o,p**)** in the metastatic IL-33-expressing tumours compared to unmodified metastatic tumour.

### The frequency of ILC2s is elevated in primary tumours and metastatic tumours expressing IL-33

**Figure 3:**
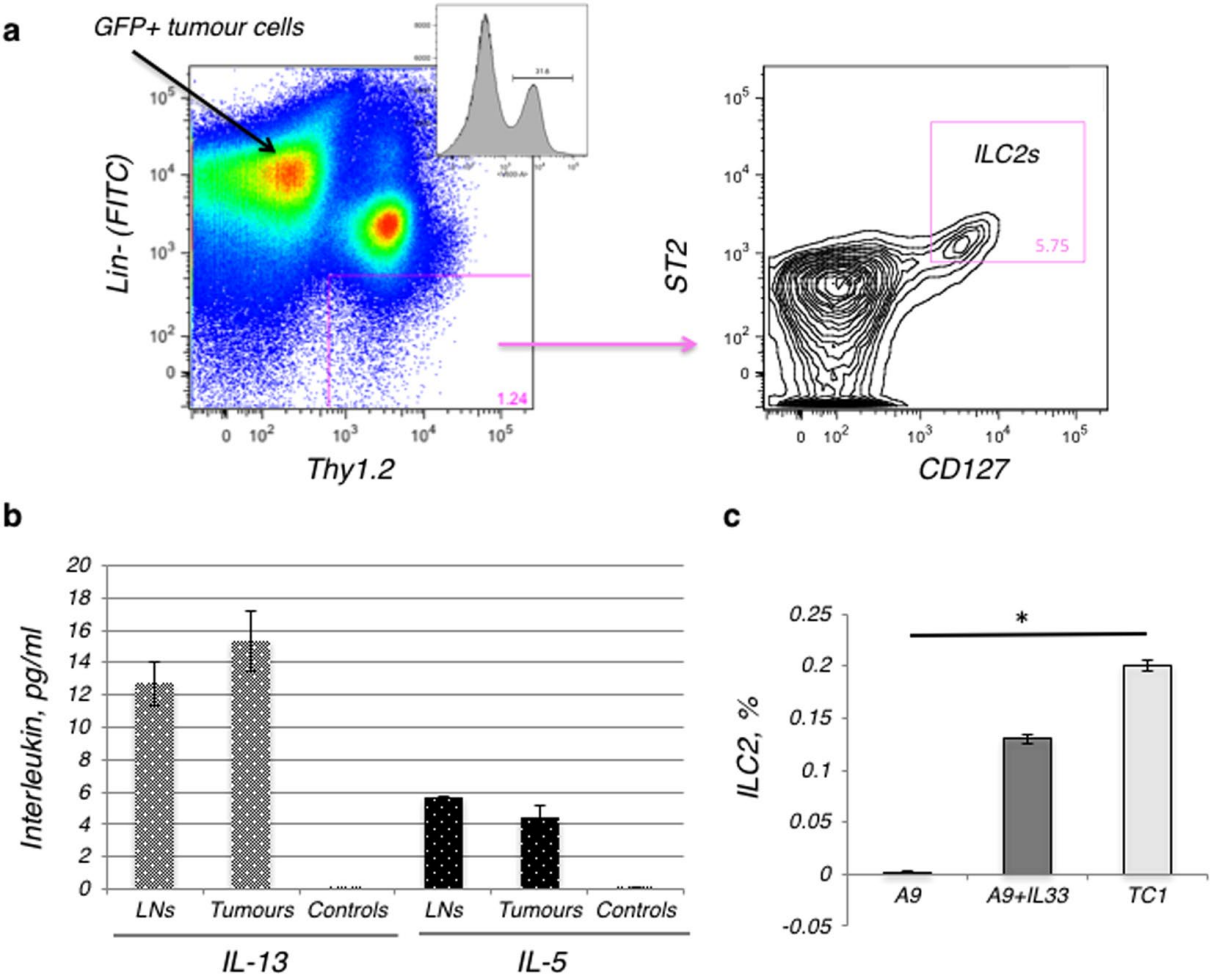
The frequency of ILC2s is elevated in primary tumours and metastatic tumours expressing IL-33, when compared to metastatic tumours not expressing IL-33. (**a**) Gating strategy: ILC2s from tumours were sorted by FACS as Lin^−^ST2^+^CD127^+^CD90.2^+^ cells. A total of 2 × 10^5^ cellular events from 1 g of disaggregated tumours were used to create a profile for each tumour. The ILC2 detection strategy included: gating on lineage-negative (Lin^−^) and Thy1.2-positive lymphocytes, with further analysis for ST2^+^ and CD127^+^ expression. The final gate (double positive for ST2 and CD127) indicates ILC2s isolated and sorted from tumour tissue, as a percentage of the Lin^−^Thy1.2^+^ cells isolated. Note, EGFP-positive tumour cells corresponded to the FITC-labeled-Lin^+^ gate and thus also appear in the “Lin^+^” gate, however these cells are not positive for Thy1.2, ST2 or CD127 and are therefore removed by subsequent gates. (**b**) Isolated ILC2s appeared to be fully functional and retained the ability to secrete IL-13 and IL-5. This graph shows ILC2s, which were isolated from either disaggregated draining lymph nodes (LN), including mesenteric, inguinal and lumbar LNs, or from TC1 tumours. Tumour cells (TC1) do not secret IL-5 and IL-13 and were used as negative controls for interleukin secretion levels. The ranges represent the data from animals within each tumour group, where lymph nodes (n = 8 animals) and tumours (n = 4 animals). (**c**) The percentage of ILC2 cells isolated from total cells in the tumour is represented in the bar graph. The number of ILC2s that could be isolated from the tumour went up in direct relation to the ability of the tumour cells to secrete IL-33. This difference was statistically significant between the number of ILC2 cells isolated from the primary TC1 and metastatic A9 tumours (*P < 0.05; Student’s t-test). Error bars represent standard error of the mean.

The difference in IL-33 expression between primary and metastatic tumours in our study model enabled examination of the involvement of ILC2s and IL-33 in cancer progression. First, we detected the presence of ILC2s in the disaggregated tumour tissue by flow cytometry, using a previously established protocol^16,26^. Briefly, ILC2s were identified as cells that did not express leukocyte lineage (Lin) cell-surface markers (Lin markers: CD3, CD4, CD8a, TCRb, CD19, CD11c, Gr-1, NK1.1, Ter119), while exhibiting a distinct pattern of cell-surface marker expression of the IL-33 receptor T1/ST2 (ST2) chain, IL-7 receptor subunit IL-7Ra (CD127) and Thy1.2 (CD90.2) (Fig. 3a). Note, EGFP-positive tumour cells corresponded to the FITC-labeled-Lin^+^ gate and thus also appear in the “Lin^+^” gate, however, these cells are not positive for either CD90.2 nor CD127 and are, therefore, removed by further gates. The population of Lin^−^ST2^+^CD127^+^CD90.2^+^ cells was demonstrated to be morphologically similar to lymphocytes: round in shape with a high nuclear to cytoplasm ratio. Upon isolation *in vitro*, this cell subset was able to grow and secrete IL-5 and IL-13 after stimulation with a combination of thymic stromal lymphopoietin (TSLP) and IL-33 **(**Fig. 3b**)**. These data indicate that the population of Lin^−^ST2^+^CD127^+^CD90.2^+^ cells detected in tumours are phenotypically and functionally tumour ILC2s.

Next, we assessed the level of ILC2 infiltration into primary (TC1) or metastatic (A9) tumours, with or without IL-33 complementation. A significant decrease in the numbers of ILC2s was observed within the disaggregated tissues of metastatic tumours (A9) versus primary (TC1) (Fig. 3c). These data suggest that ILC2s are involved in anti-tumour immune surveillance.

### Demonstration that ILC2s aid in immune recognition of cancers *in vivo*

To directly examine the role of ILC2s and IL-33 in cancer development, we conducted a comparison of the progression of the primary (TC1) or metastatic (A9) tumours, with and without IL-33-gene complementation, in mice transplanted with bone marrow (BM) from either wild type (WT) mice or mice lacking ILC2s (RORα^−/−^). RORα^−/−^ mice have severe neurological defects and do not survive long past weaning, however, RORα^−/−^ BM-transplanted mice are ILC2-deficient while retaining functional immune cells including Th2 cells and RORγt^+^ ILCs^27^. Therefore, BM chimeras were generated by reconstitution of lethally irradiated B6.Pep3b (CD45.1) mice with whole BM cells from either 4-week-old WT or RORα^−/−^ (both CD45.2) mice. BM transplant recipients were allowed to recover for 6 weeks, and the quality of the BM transplantation was analysed by FACS, determining the ratio between CD45.1 and CD45.2 positive cells in peripheral blood. All the transplants were between 92–96% efficient (Supplemental Fig. 1). As a result, chimeric mice transplanted with WT (C57Bl/6) BM were able to produce WT levels of ILC2s, and chimeric mice transplanted with RORα^−/−^ BM were able to produce only miniscule numbers of ILC2s (resulting from the residual B6.Pep3b host background). Note, in the mice transplanted with RORα^−/−^, BM only the BM was RORα^−/−^ with the rest of the mouse remaining WT, meaning that only ILC2s were lacking in the mice transplanted with RORα^−/−^ BM. A9, A9+IL-33, or TC1 cells were then injected s.c. into the flank of both the mice transplanted with WT BM or RORα^−/−^ BM, and allowed to grow.

**Figure 4:**
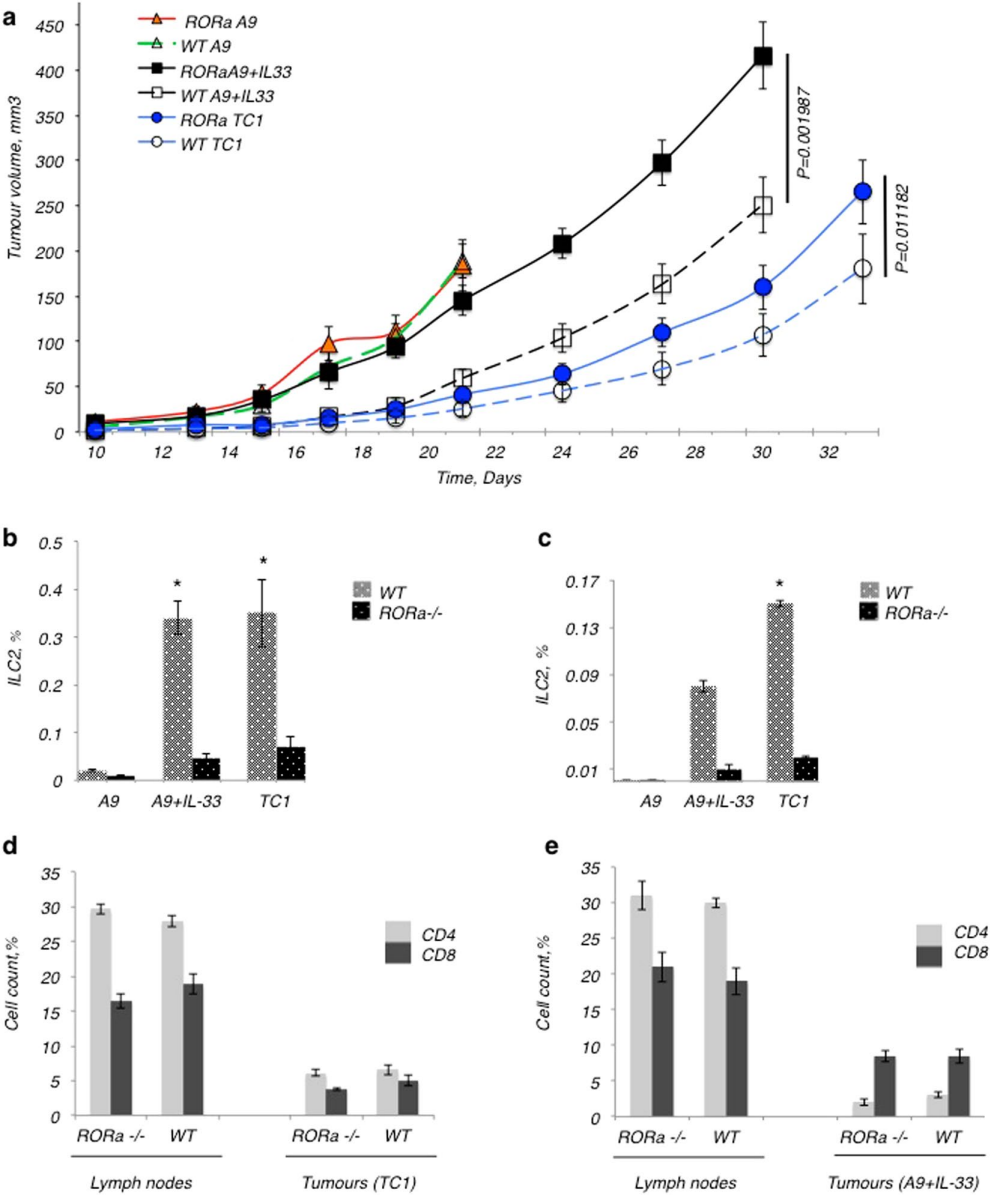
The mice lacking ILC2s (RORα^−/−^) were less able to limit the growth of tumours. Tumours with and without IL-33-expression were established in the WT and RORα^−/−^ chimeric mice. (**a**) The presence of ILC2s significantly inhibited tumour formation in WT chimeric mice bearing tumours expressing IL-33, when compared to RORα^−/−^ chimeras, which lacked ILC2s. Metastatic A9 tumours demonstrated rapid progression and severe disease symptoms reaching the humane end-point that resulted in early termination of animals from both A9 chimeric groups, indicating that the ILC2s were not significantly able to control the growth of fast growing tumours which lack IL-33 expression. Metastatic A9+IL-33 tumour growth was more aggressive in RORα^−/−^ chimeric mice lacking ILC2s, to the point where they resembled the growth of A9 cells alone. The difference in growth results between metastatic A9+IL-33 tumours grown in WT versus RORα^−/−^ chimeric mice was significant; P < 0.002 (Student t-test). Primary TC1 tumours were able to grow faster in mice lacking ILC2s (RORα^−/−^ chimeras) than in WT chimeras P < 0.05 (Student t-test). (**b**) The numbers of ILC2 cells found in neighbouring lymph nodes were significantly lower in RORα^−/−^ mice compared to WT chimeras bearing IL-33 expressing tumours. This served as a quality control for bone marrow transplantation; *P < 0.05 (Student t-test). (**c**) The numbers of ILC2 cells found in primary TC1 tumours were significantly higher than in metastatic A9 or A9+IL-33 tumours; *P < 0.05 (Student t-test). (**d,e**) RORα deficiency had no effect on the percentage of CD4^+^ or CD8^+^ lymphocytes found in either lymph nodes or tumours in response to (**d**) primary TC1 or (**e**) metastatic A9+IL-33 tumours. The percentage of all cells was calculated as a fraction of 2 × 10^5^ cellular events used to create a profile for each organ or tissue. The error bars represent standard error of the mean; n = 8 mice per group.

Overall, the ability of the mouse to produce ILC2s resulted in significantly reduced tumour growth over time (Fig. 4). The frequency of ILC2s isolated from lymph nodes of WT animals bearing IL-33-expressing tumours (*i.e*, TC-1 or A9+IL-33) was significantly increased over animals bearing tumours not expressing IL-33 (*i.e*. A9 alone) (Fig. 4b). The frequency of ILC2s isolated from lymph nodes of mice transplanted with RORα^−/−^ BM bearing IL-33-expressing tumours was less than 0.05% of total cells isolated, and further served as a control for the efficiency of bone marrow transplantation. Accordingly, the frequency of ILC2s isolated from resected IL-33 expressing tumours from mice transplanted with WT BM was consistently higher (Fig. 4c), when compared to tumours not expressing IL-33. Strikingly, the number of ILC2s isolated from IL-33-expressing metastatic A9 tumours was more than 30-fold higher than what could be isolated from metastatic A9 tumours not expressing IL-33 in mice transplanted with WT BM. This increase in the numbers of ILC2s paralleled the reduced growth rate seen by both the primary TC1 or metastatic A9+IL-33 tumours, as compared to the metastatic A9 tumours in mice transplanted with WT BM (Fig. 4a). The expression of IL-33 by metastatic A9 did not appear to change the growth rate in animals lacking ILC2s however, *i.e*. mice transplanted with RORα^−/−^ BM. This implies that the presence of IL-33 in the tumour microenvironment increases the ILC2 frequency and activity in the tissue of the mice transplanted with WT BM, affecting the tumour growth rate accordingly.

We did not detect ILC2 cells in IL-33-deficient metastatic tumours (A9) established in either the mice transplanted with WT BM or RORα^−/−^ BM (Fig. 4c). The animals from these groups were terminated earlier (on Day 21) due to severe clinical presentation (Fig. 4a), including ulceration of tumours. The lack of expression of IL-33 in the metastatic A9 tumours likely contributed to their inability to support or attract ILC2s to the site of the tumour; and consequently, the microenvironment of the metastatic A9 tumours does not appear to support the development and function of ILC2s. This is further supported by the observation that ILC2s were also not found in significant numbers in the draining lymph nodes surrounding the metastatic A9 tumours in mice transplanted with WT BM, whereas they could be detected in lymph nodes surrounding primary TC1 tumours and metastatic A9+IL-33 tumours (Fig. 4b).

### The role of ILC2 immune cells in limiting metastasis

**Figure 5:**
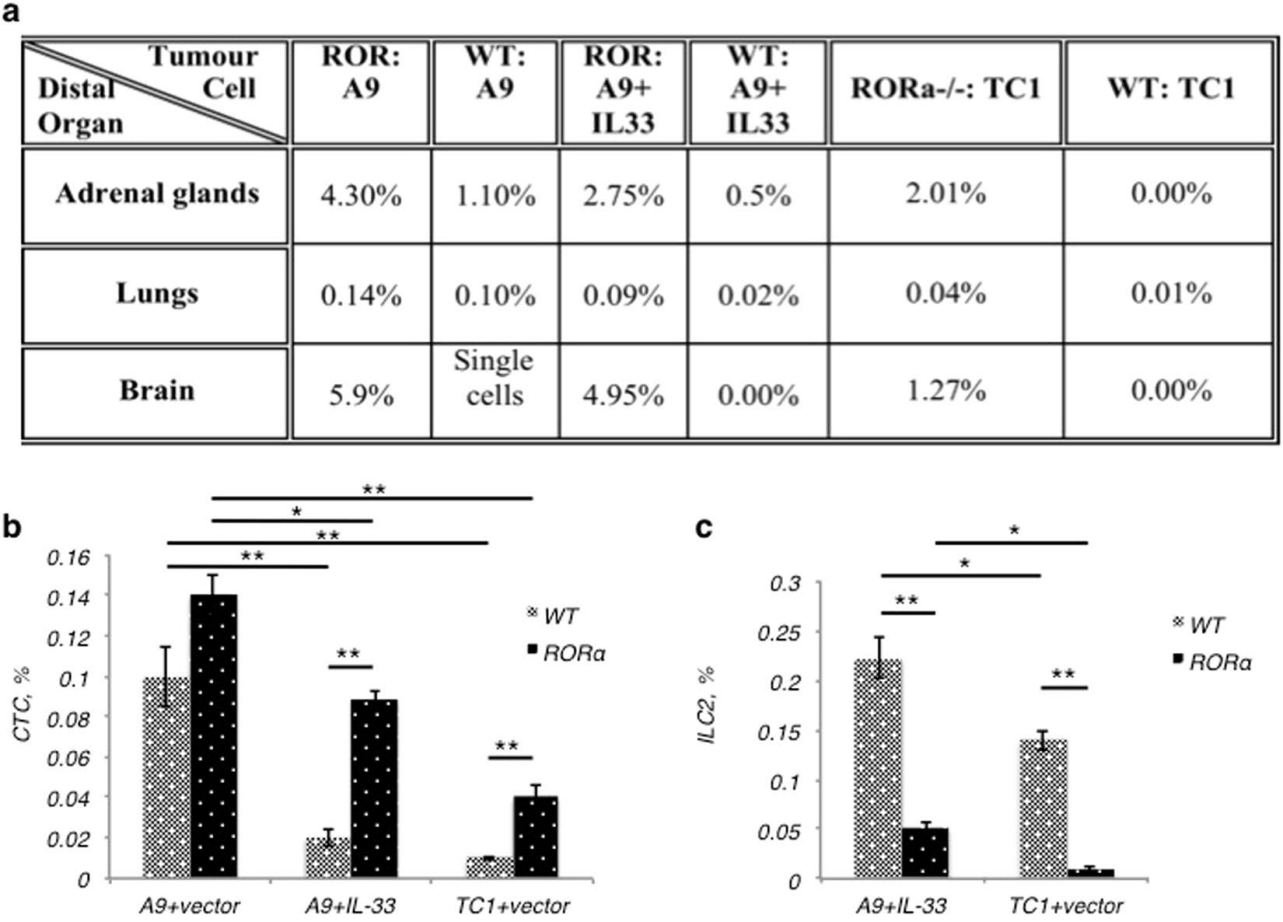
Functional ILC2 cells are important in controlling the metastatic spread of the tumours. (**a**) Spread of EGFP-positive circulating tumour cells (CTC) to distal organs was higher in chimeric mice lacking ILC2s (RORα^−/−^ mice); even primary TC1 cells that are not metastatic in the WT mice could be found beyond the initial site of transplantation in mice lacking ILC2s. (**b**) In lungs, CTC counts were higher in animals with reduced number of ILC2s (RORα^−/−^). (**c**) The number of ILC2s present in the lungs appeared to go up with the presence of CTCs in the lungs. The percentage of all cells was calculated from the fraction of live cells in 2 × 10^5^ events used to create a profile for each organ; n = 8 mice per group; *P < 0.05; **P < 0.01.

To test the involvement of ILC2 innate immune cells in the metastatic spread of tumours, we assessed the frequency of tumour cells in common metastatic sites for lung carcinoma. Stable transfection of tumour cells with pIRES2-EGFP allowed us to track the appearance of EGFP-positive CTCs in distal organs, including adrenal glands, lungs, and brain. Note, the initial site of tumour establishment was subcutaneously in the right flank; the tumour cells were never given intravenously. We detected higher percentage of EGFP+ cells in all distal organs isolated from RORα^−/−^ mice compared to WT mice (Fig. 5a). Furthermore, the spread of metastatic A9 tumour cells was higher when compared to metastatic A9+IL-33, in both WT and RORα^−/−^ bone marrow chimeric mouse models. Most importantly, we observed that while the presence of ILC2s was not able to significantly reduce the overall rate of growth of A9 metastatic tumours locally (Fig. 4a), they were critical in reducing the metastatic spread of these metastatic A9 tumour cells (Fig. 5a). We detected an increase in metastatic A9 CTCs up to 4-fold in adrenal glands, 1.5-fold in lungs, and more than 1000-fold in brains resected from mice lacking ILC2s (mice transplanted with RORα^−/−^ BM). This is an impressive finding and suggests for the first time, that ILC2s can function in dramatically limiting metastasis. Furthermore, in contrast to mice transplanted with WT BM, we unexpectedly detected the formation of metastases from the primary TC1 tumours in ILC2-deficient mice transplanted with RORα^−/−^ BM. We detected a considerable frequency of CTCs in adrenal glands, lungs and brains (Fig. 5a) of mice transplanted with RORα^−/−^ BM that bore primary TC1 tumours, even though we have shown in Table 1, that this cell line is not predisposed to move to distal sites in WT animals. Overall, these data indicate that ILC2s are important for controlling metastatic spread of both primary and metastatic cancer cells. Furthermore, the presence of IL-33 in the microenvironment augments the anti-cancer function of ILC2s.

### Mobilization of ILC2 immune cells from the lung

To test whether the spread of the tumour cells to the lungs may alter the level of local lung ILC2s, we quantified lung ILC2s from mice bearing either metastatic A9+IL-33 or primary TC1 tumours. Note that in animals bearing metastatic A9 tumours alone, the tumour-associated ILC2 numbers were essentially absent or at least too low to quantitate. The number of CTCs appeared to be directly correlated with the number of ILC2s that could be isolated. Thus, the higher number of CTCs (Fig. 5b) resulted in a correspondingly higher number of ILC2s found in the organ (Fig. 5c). We hypothesize that the presence of CTCs in lungs stimulates the local immune response, increasing the recruitment of lung ILC2s. In contrast, the suppression of ILC2 number and function in mice transplanted with RORα^−/−^ BM allowed the metastases to increase, suggesting the importance of the RORα transcription factor and the presence of functional ILC2s for the proper development of anti-tumour immune responses. Collectively, these data support the conclusion that the presence of ILC2s is important in limiting metastases and that ILC2s participate in cancer immune-surveillance through an IL-33/ILC2 axis.

### Evaluation of the functionality of lymphocytes from RORα^−/−^ mice

**Figure 6:**
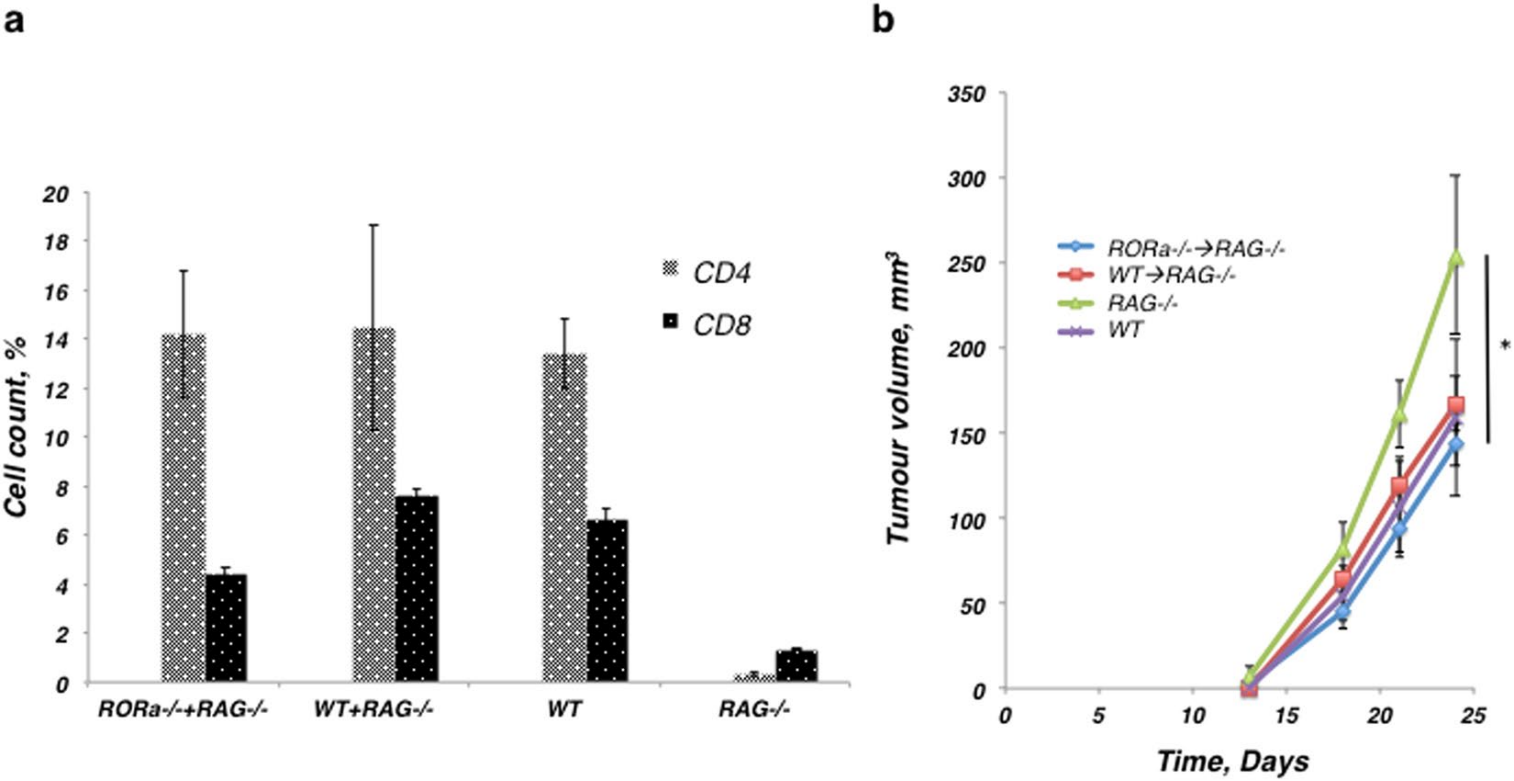
RORα^−/−^ mice produce functional lymphocytes. **(a)** Immune cell count in the peripheral blood of (WT → RAG^−/−^) and (RORα^−/−^ → RAG^−/−^) animals, as well as in the WT and RAG^−/−^ control animals. **(b**) The T and B cells isolated from the RORα^−/−^ and WT mice were equivalent in their functional ability to suppress the tumour growth to the level of the WT control group, however, the tumour growth was significantly higher in RAG^−/−^ control group when compared to the (RORα^−/−^ → RAG^−/−^) group of mice (*P = 0.29, Student t-test). The percentage of all cells was calculated as a fraction of 2 × 10^5^ cellular events used to create a profile for each organ or tissue. The error bars represent standard error of the mean; n = 8 mice per group.

In order to complement the previous experiment shown in Fig. 4 where we demonstrate that the lack of ILC2s results in higher tumour growth and metastasis, we sought to confirm that this mechanistic effect was indeed due to a lack of ILC2s and not due to the lack of functional lymphocytes in the RORα^−/−^ mice. We undertook this by reconstituting RAG^−/−^ mice (which lack T and B cells, but produce ILC2s) with splenocytes from either RORα^−/−^ (which lack ILC2s, but produce T and B cells) or WT mice. The RAG^−/−^ mice were not irradiated and their ILC2s remained functional, and the RORα^−/−^ splenocytes that were transferred, had developed in a RORα^−/−^ environment. Four weeks later we analyzed the peripheral blood of transplanted animals and the control mice (WT and RAG^−/−^). Comparing the numbers of CD4^+^ and CD8^+^ T cells in peripheral blood of WT → RAG^−/−^ and RORα^−/−^ → RAG^−/−^ mice, we found that cell numbers in T cell compartments were similar for all groups (Fig. 6a), except the RAG^−/−^ negative control which showed minimal lymphocyte presence. We then established TC1 tumours in the four groups of mice: RAG^−/−^ alone; WT alone; WT → RAG^−/−^; and RORα^−/−^ → RAG^−/−^. We found there was no statistical difference in tumour growth between WT alone and WT → RAG^−/−^ (p = 0.799429), WT alone and RORα^−/−^ → RAG^−/−^ (p = 0.529102), WT → RAG^−/−^ and RORα^−/−^ → RAG^−/−^ (p = 0.449731) mice, indicating that the T and B lymphocytes received from the RORα^−/−^ and WT mice were equivalent in their functional ability to limit TC1 tumour growth (Fig. 6b). Collectively, these data demonstrate that ILC2 cells contribute to the ability of lymphocytes to mediate an immune response against tumours and, reciprocally, lymphocytes require ILC2s to carry out this function.

### Effect of ILC2s on CTL effector mechanism

**Figure 7:**
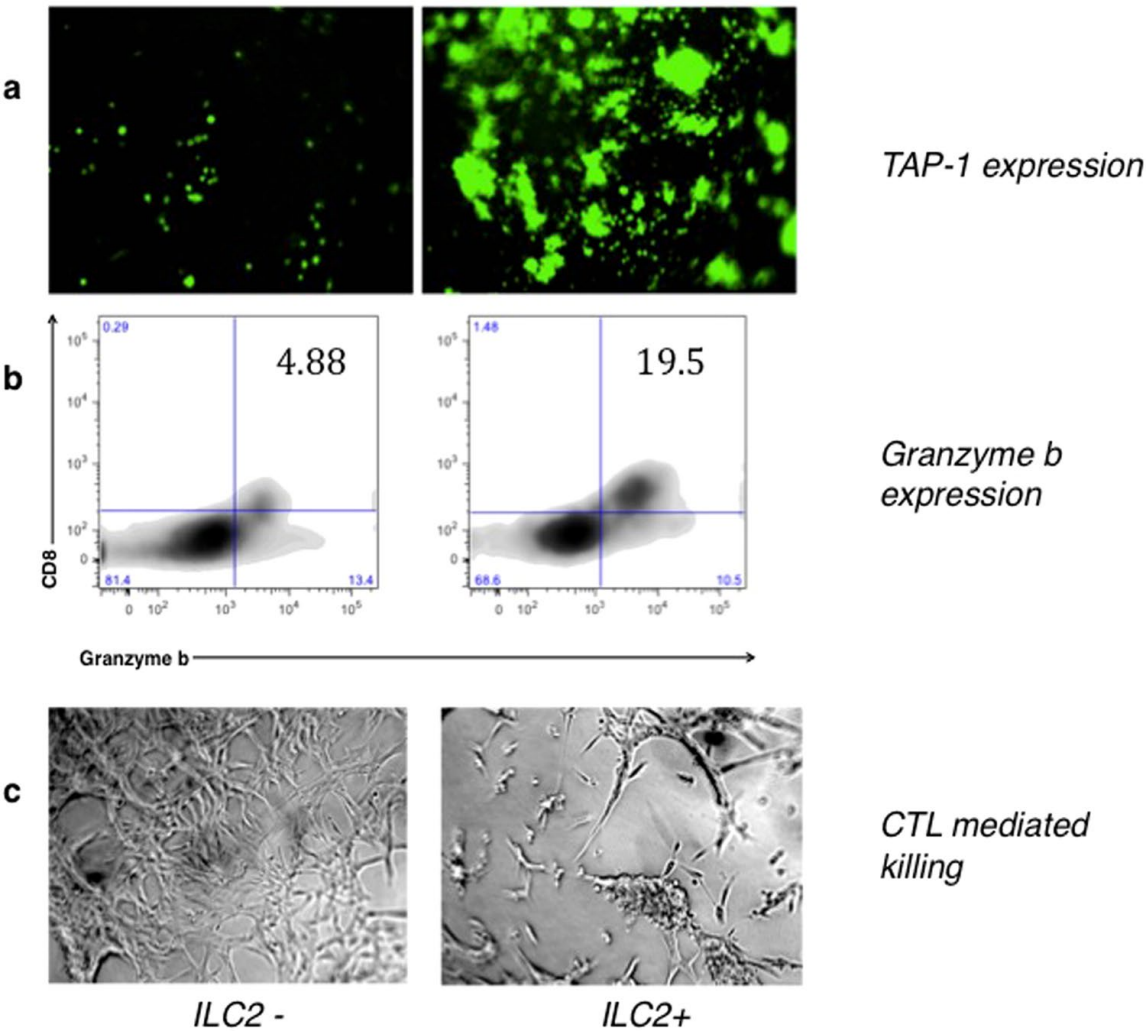
Impact of ILC2s on specific CTL effector mechanisms. Co-culture of metastatic murine prostate TAP-1-low carcinoma cells (LMD) and CD8 T cells with ILC2s (right panel) or without ILC2 cells (left panel): (**a**) TAP-1 expression level in LMD cells after activation with ILC2 cells (right) and without ILC2 cells (left); (**b**) Granzyme b expression by CTL cells in the absence of ILC2s (left) or in the presence of ILC2s (right); (**c**) Light microscope images of cell co-cultures after the incubation with CTLs show the extent of CTL-mediated killing, either in the presence or absence of ILC2s.

To assess the effect of ILC2s on CTL effector mechanisms, ILC2 were isolated from tumours of donor-mice, three weeks after initial tumour inoculation. The isolated ILC2s were then activated *in vitro* with IL-33 and TSLP. Subsequently metastatic prostate tumour cells (LMD, described previously^21,23^) were cultured with and without activated ILC2s. 48 hours later CD8^+^DCs were added and the cellular mixture was pulsed for ~4 h with OVA (257–264) peptide. Freshly isolated CD8a^+^ OT-1 T cells were then added to the wells, and all cells were incubated together for four days. The metastatic LMD prostate tumour cells express EGFP under the transcriptional control of the TAP-1 promoter^28^, where an increase in EGFP expression correlates with an increase in both TAP-1 and MHC-I expression, and where elevated level of these components is associated with higher recognition of the tumour cells by T cells. The presence of activated ILC2s in the system elevated the EGFP expression of the metastatic LMD cells, indicating the LMD cells have been stimulated to express MHC I antigen presentation molecules on their surface. This observation suggests that the direct interaction with the ILC2s or with cytokines secreted by them enables the metastatic LMD cells to overcome their antigen presentation deficiencies (Fig. 7a). The elevated level of Granzyme b in the CD8a^+^ T cells (Fig. 7b) and the increased cellular killing seen by microscopy (Fig. 7c) further indicates that the CTL effector mechanisms have been heightened in the presence of ILC2s, providing a mechanism for ILC2 action in anti-cancer immunity.

### Differential gene expression between primary tumours, metastatic tumours or metastatic IL-33-expressing tumours

**Figure 8:**
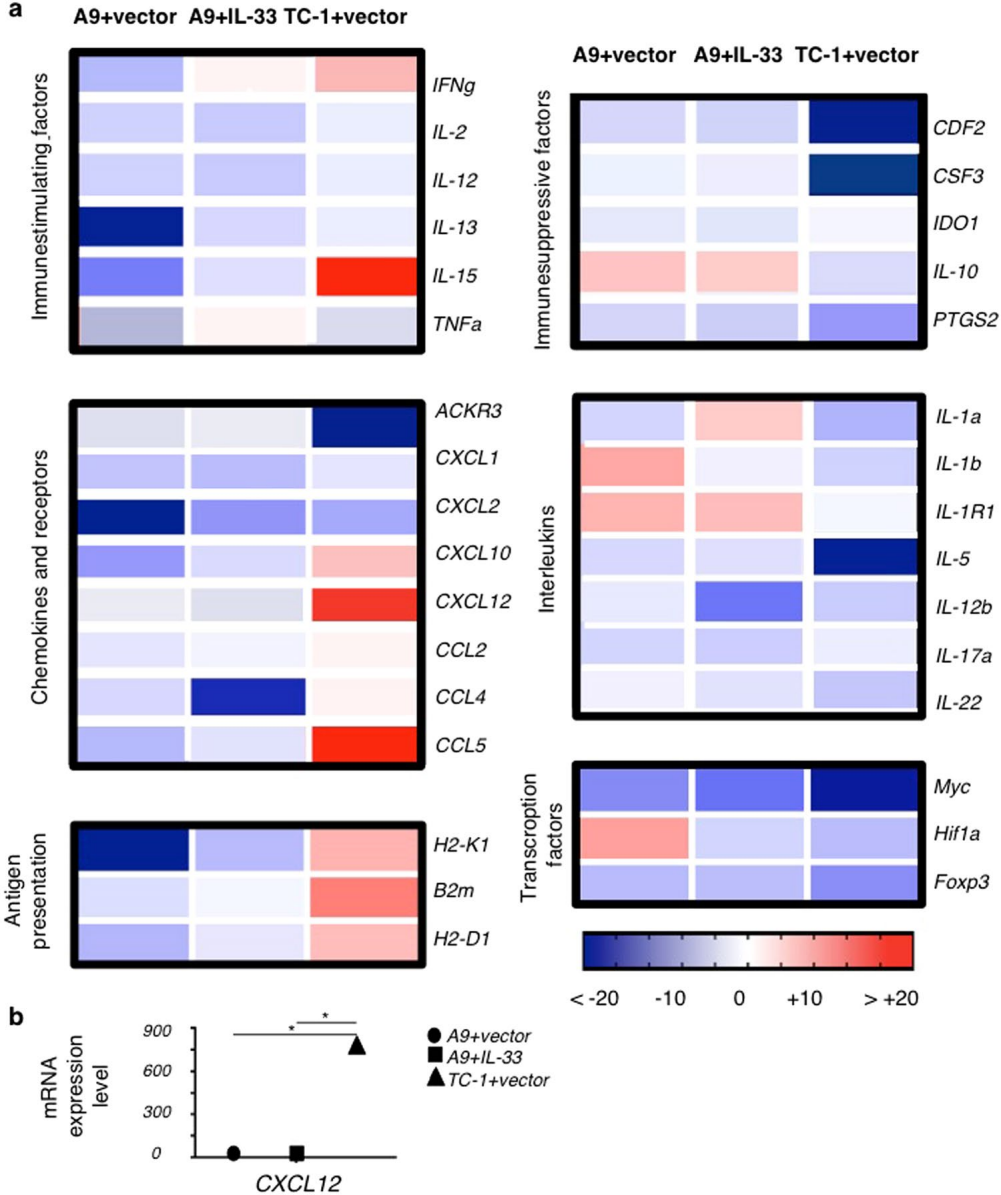
Heat map illustrates the relative expression of genes in metastatic A9+vector cells, metastatic A9+IL-33 cells and primary TC1+vector cells. The Mouse Cancer Inflammation & Immunity Crosstalk RT^2^ Profiler PCR Array was used to analyze cDNA from the tumour cell lines grown *in vitro*. Relative changes in gene expression were calculated using the ΔΔCt (threshold cycle) method. Changes in cycle threshold (ΔCt) values for each tested gene were obtained by subtracting the average of the threshold cycle (Ct) values for the housekeeping genes (Gusb, Hsp90ab1, Gapdh, and Actβ) from the threshold cycle value of the gene. The expression fold change was calculated as (2^(−ΔΔCt)^). Expression of the housekeeping genes between the tumour cell samples (metastatic A9+vector; metastatic A9+IL-33; primary TC1+vector) were normalized to the transcription levels seen in the metastatic A9+vector samples. The ratio up- or down-regulation was then calculated relative to the normalized transcription of each gene in each of the tumour cell lines. (**a**) Immunostimulatory factors are down-regulated in metastatic cells (A9), whereas factors involved into immunosuppressive pathways are reduced in primary tumours (TC1). Antigen presenting components are over expressed in primary tumours that is well in line with up-regulation of chemo-attractants and with a better anti-tumour immune response. (**b**) The level of CXCL12 expression is almost 1000-fold higher in IL-33 expressing primary tumour cells (TC1) and metastatic cells (A9+IL-33) when compared to metastatic tumour cells (A9 alone), P < 0.01 (Student t-test).

To obtain insights into the changes in gene expression that take place as a result of IL-33 expression by tumour cells, we conducted a pathway-focused gene expression analysis, using the “Mouse Cancer Inflammation & Immunity Crosstalk RT^2^ Profiler PCR Array” (Qiagen). We quantitatively analyzed RNA-expression from the tumour cells and identified distinct gene expression signatures in the different samples: A9 (metastatic tumours), A9+IL-33 (metastatic), and TC1 (primary tumours) (Fig. 8). Metastatic and primary tumours exhibit differences in the expression of immunostimulatory and immunosuppressive factors, antigen presenting molecules, interleukins, chemokines and chemokines receptors, Toll-like receptors (TLRs) and transcriptional factors. TC1 primary tumour cells had higher levels of several immunostimulatory factors (IFNγ, IL-2, TNFα), including antigen presenting molecules (H2-K1) as compared to the A9 metastatic tumour cells. This may explain why the A9 cells appear to proliferate unchecked by the adaptive immune response during cancer progression. These findings were reinforced by the lower expression of immunosuppressive components (Ido, IL-10, Ptgs2) in TC1 primary tumour cells^29,30^, creating a less favourable microenvironment for tumour cell survival and proliferation. Interestingly, the metastatic and primary tumours expressed different TLR genes. TLR3 was selectively up-regulated by TC1 primary tumour cells and this was corroborated by enhanced expression of genes associated with the IFNγ signaling pathway^31^. Activation of TLR3 signaling leads to IFN type I production, which is usually associated with innate immune response. Thus, type I^32^ and type II^33^ interferons together with IL-33 may regulate ILC2 immunopathology. We also found that T cell chemoattractants are significantly lower in A9 metastatic tumour cells, when compared to metastatic A9+IL-33 and primary TC1 tumour cells (Fig. 8). Impaired migration towards CCL5^34^ and CXCL12^35^ may reduce the access of T cells to the tumour tissues, facilitating A9 tumour cell survival and progression. Transcriptional factors, which are typically associated with this process (Myc, Foxp3), were more highly expressed in metastatic cells when compared to primary tumour cells, highlighting the differential transcriptional processes of primary and metastatic tumour cells. The PCR array also included mediators and effectors of the cross-talk between tumours and the immune system that influences the course of tumour development. Thus, the results support the conclusion that changes in the expression of tumour genes affect the immune response and this plays an important role in the evolution of primary tumours into the immunosubversive metastatic form.

Given that ILC2 are developmentally and functionally dependent on the presence of IL-33, we compared the transcriptional profile of metastatic tumour cells before and after genetic modification with IL-33 gene. We found that components of the antigen processing pathway (H2-K1, β2 m) were elevated in A9 metastatic tumours expressing IL-33, whereas the expression of immunosuppressive factors (IL-10, Ptgs2) were diminished, thereby altering the tumour microenvironment toward immune responsiveness. Furthermore, a variety of immune chemoattractants were found to be increased in metastatic A9+IL-33 cells when compared to metastatic A9 alone (Fig. 8): CXCL2 (attracting neutrophils); CCL5 (attracting macrophages); and CSCL10 (attracting NK and CD8+T cells). These chemoattractants are secreted by the IL-33 expressing tumour, and have been previously shown to create a permissive environment for infiltration of the immune cells^36–39^. The resulting changes in gene expression induced by IL-33 enables host to overcome the immunosuppressive state to constrain invasion and metastasis. Re-expression of IL-33 in metastatic cells allows for the attraction of innate and adaptive immune cells, and allows the host to orchestrate more efficient immune responses.

## Discussion

This is the first study to demonstrate the direct involvement of ILC2s in the immune recognition and elimination of tumours. Furthermore, we observe that subversion of ILC2-mediated immunity against tumours takes place concomitantly with the transition from primary to metastatic tumours and the concurrent reduction of ILC2s represents an entirely new form of tumour immune-escape.

ILC2s have a number of parallels with that of CD4^+^ T helper (Th) cells. The development of both ILC2s and CD4^+^ Th cells have a requirement for the same transcription factor, GATA3^40,41^, and have similar profiles of secreted cytokines, *e.g*. IL-13 and IL-5. However, even though there is biochemical redundancy between the cells, there are a number of other factors that are contrasting between these cells; including differential regulation of each cell type regarding the production of the same cytokines^42,43^, context-specific nature of the stimuli^44^, and intensity of paracrine signalling between normal and neoplastic cells in the microenvironment, which is also associated with the stage of the disease. Further studies are required to better understand the interactions between various players of the innate and adaptive immune systems in the tumour microenvironment. In particular, to identify how a common set of transcriptional regulators shared by developmental programming of ILCs and T cells may enable them to acquire associated effector functions.

Initially, the difference in IL-33 levels within the tumour microenvironment of the primary and metastatic carcinomas was the clue that led us to study ILC2s, whose development and function are strongly dependent on IL-33 expression^11,19,45^. A significant decrease in the ILC2 number in metastatic tumours versus primary, or IL-33-complemented tumours, was observed, providing further evidence that ILC2s could contribute to tumour immune surveillance. We next found that the largest tumours were formed from metastatic tumour cells that had little to no IL-33 expression and these corresponded to tumours from which little to no ILC2s could be isolated. These data suggest that ILC2s are involved in immune-surveillance towards tumours that led us to ask whether cancer growth and metastasies would be more aggressive in the absence of ILC2s.

The generation of bone marrow chimeras made it possible to study the mechanistic details of the involvement of ILC2s in the modification of tumour progression. We transplanted RORα^−/−^ bone marrow into mice of a WT background, resulting in mice that lack ILC2s^27^, but which maintained wild type levels of other lymphocytes. In the resulting ILC2-deficient mice (mice transplanted with RORα^−/−^ BM), we observed that IL-33-expressing primary tumours (TC1) or IL-33 expressing metastatic tumours (A9+IL-33) grew more rapidly and had a higher frequency of metastasis in ILC2-deficient mice, whereas the growth rate of the A9 cells not expressing IL-33 was not found to be significantly different between mice with or without ILC2s (Fig. 4). This indicates that the tumour microenvironment is important for ILC2-mediated cancer immune-surveillance, and in particular the expression of IL-33 by the tumour may allow the activation of ILC2 function, whereas the lack of IL-33 expression would not support these immune cells.

An important observation was a dramatic increase in the ability of the primary and metastatic tumours to metastasize in the absence of ILC2s (Fig. 5). While the local growth of the metastatic A9 tumours lacking IL-33 expression at the site of injection was not significantly different between either the ILC2-deficient mice (transplanted with RORα^−/−^ BM) or ILC2-producing mice (transplanted with WT BM), the numbers of A9 CTCs were dramatically increased in mice lacking ILC2s. Furthermore, a similar increase in metastases was noted in ILC2-deficient mice for metastatic A9+IL-33 tumours, and most strikingly, for the primary TC1 tumours, which are not predisposed to form metastases in any setting previously studied. These data suggest that ILC2s are directly involved in the cellular immune-surveillance that limits tumour growth and potential to metastasize and colonize distal organs^6,8,11–13^, possibly, due to direct correlation between ILC2s and E-cadherin in an IL-33 dependent manner^46^.

We addressed whether the effects on tumour growth within the RORα^−/−^ mice were due to the lack of ILC2s and not due to the lack of functional T or B cells. To do so, we analyzed the growth of TC1 tumours in RAG^−/−^ mice (lacking T and B cells) that had been supplemented with splenocytes obtained from either WT or RORα^−/−^ animals (WT → RAG^−/−^ and RORα^−/−^ → RAG^−/−^) and compared them to the growth of TC1 tumours in WT or RAG^−/−^ control animals (Fig. 6). We found the similar level of tumour growth reduction in the WT, WT → RAG^−/−^ and RORα^−/−^ → RAG^−/−^ animals, as compared to the tumour growth level in the RAG^−/−^ only mice. These results were consistent with the similar levels of CD4+ and CD8+T lymphocytes in peripheral blood of WT, WT → RAG^−/−^ and RORα^−/−^→RAG^−/−^ animals, and their extremely low levels in the RAG^−/−^ only mice. Recent studies on the spectrum of lymphocytes developed in RORα-deficient conditions have supported our observations. Halim *et al*.^27^ demonstrated that mice transplanted with RORα^−/−^ bone marrow specifically lack ILC2s, but maintain normal numbers of NK cells (ILC1s), ILC3s, CD4 and CD8 cells and Th17 cells^27^. They concluded that in BM chimeras that RORα-deficiency had no effect on the development of other lymphocytes, and that specifically RORγ^+^Th17^+^ were not significantly different between mice transplanted with either RORα^−/−^ BM or WT BM. Collectively, these data demonstrate that ILC2 cells facilitate lymphocytes to mediate the adaptive immune response against tumours and, reciprocally, lymphocytes require ILC2s to carry out this function.

To highlight the functional enhancement in the T cell compartment, we demonstrated elevated levels of cytolytic T cell effector mechanisms, including the elevated level of Granzyme b production by CD8+T cells and enhanced cellular killing, after the direct interaction of murine metastatic prostate cancer cells with CD8+T cells and the activated ILC2 cells (Fig. 7). Moreover, the increased number of immune cells within primary tumours or metastatic IL-33-expressing tumours (Fig. 2) supports the conclusion that tumour-infiltrating innate and adaptive immune cells can overcome immune-deficiency in metastatic tumours *in vivo*. Overall, our data supports the conclusion that the high level of IL-33 provides immune stimulatory microenvironment that is directly correlated with the presence of the innate and adaptive immune cell subsets that mediate protective anti-tumour immunity.

ILC2 cells have been distinguished in the literature as a predominant source of IL-13 *in vivo*^9,10,47^. Consistent with this identification, ILC2 cell subset has been associated with eotaxin production and eosinophil recruitment^16,48,49^. In an immune stimulatory tumour microenvironment, eosinophils would produce high numbers of potent chemoattractants for CD8+T cells via STAT1, such as CCL5, CXCL10, CXCL11^18^. Whereas, in an immune suppressive environment, eosinophils would employ STAT6 to produce CCL17, CCL22 that are known to attract Th2 cells and promote macrophages to develop into a “Type 2” or a “Tumour-associated” phenotype, which are known to be less active against tumours than “Type 1” macrophages^50,51^.

**Figure 9:**
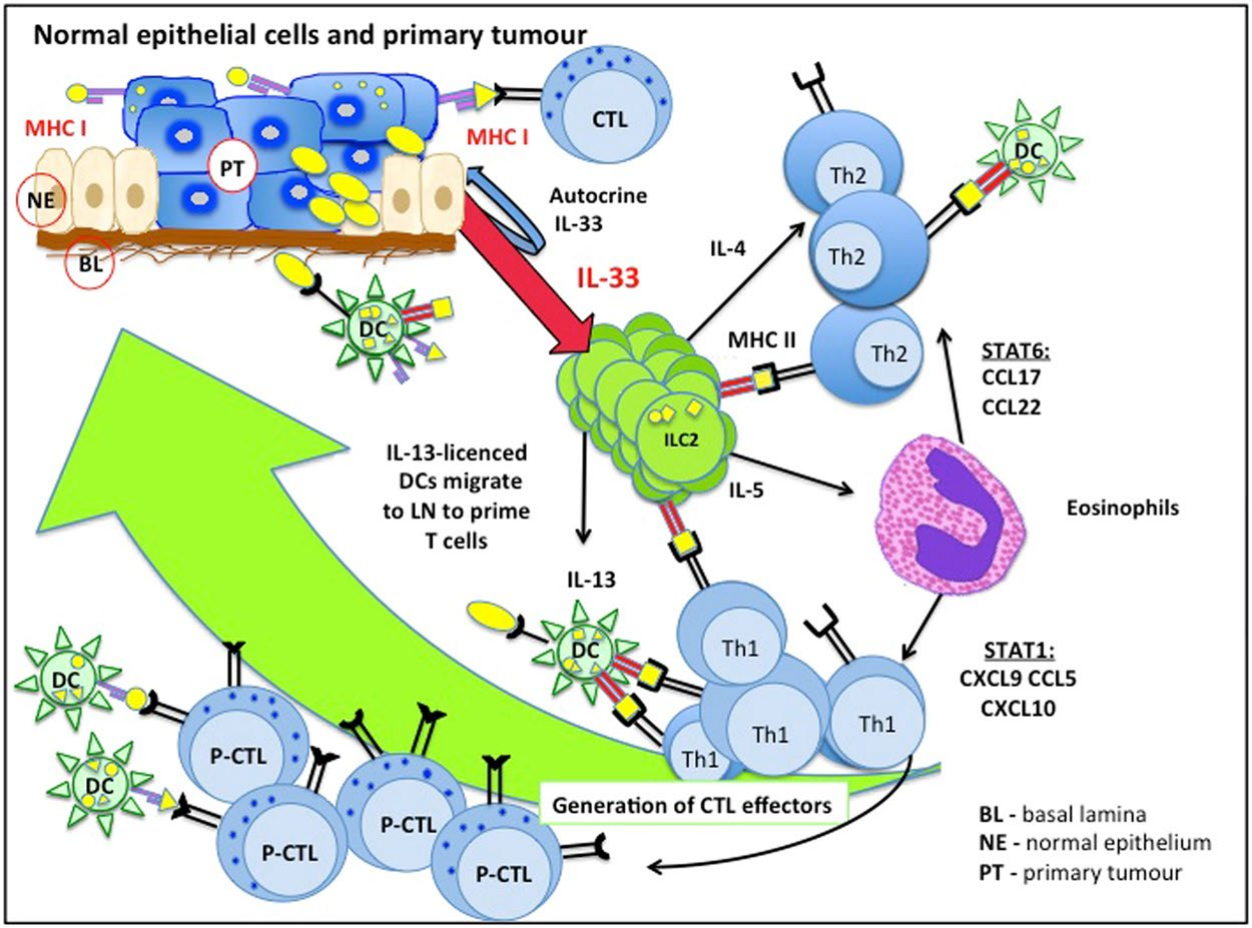
Model linking the adaptive and innate immune responses during tumour development via IL-33-ILC2 axis. IL-33-expressing tumour environment stimulates the development of ILC2 cells and functionally activates them through the ST2 receptor pathway. Functionally active ILC2s alter the tumour microenvironment triggering both innate and adaptive immune responses. ILC2s recruit DCs through IL-13 production, stimulate Th2 cells and possibly Th1 cells through direct ILC2 antigen presentation via MHC-II molecules, and indirectly stimulate CTL precursor cells through DC endogenous antigen presentation or cross-presentation via MHC-I molecules. Th1 and Th2 cells may also be activated by DCs through exogenous antigen presentation via MHC-II molecules. Through the release of IL-5 by ILC2s and subsequent recruitment of eosinophils, the chemokine profiles of tumour microenvironment is changed to attract Th1 and CD8+ T cells and to direct the activation of CTL-mediated killing and cancer rejection. This mechanism acts to suppress the frequency of circulating tumours cell and subsequent metastasis. In metastatic tumours with low IL-33 content, the IL-33/ILC2 pathway is not initiated and immune escape is facilitated.

In our previous paper (Saranchova *et al*.)^21^, we demonstrated that the presence of IL-33 in the system alters the tumour microenvironment towards a proinflammatory state. Here we show that not only are the components of the antigen processing pathway (H2-K1, β2 m) elevated in IL-33 expressing tumours, but also are a variety of chemoattractants (CCL5, CXCL10, CXCL12) that coordinate the transportation of the innate and adaptive immune cells to the tumour site, whereas the production of immunosuppressive factors (IL-10, Ptgs2) is reduced. Taking together, the resulting changes in gene expression induced by IL-33 are able to modify the tumour microenvironment and to attract more innate and adaptive immune cells in order to orchestrate anti-tumour immune responses. The increased number of immune cells in IL-33 expressing tumours was observed in mouse and in human tumour tissues. Thus, the IL-33/ILC2 axis is a missing link between the adaptive and innate immune responses during tumour development (Fig. 9).

It is important to point out that current literature is somewhat controversial regarding the role of IL-33 in tumour development. For example, in contrast to our studies, Wang *et al*., (2016) teaches that IL-33 signaling fuels the growth of human lung cancer, however, the authors only examined IL-33 expression in isolated primary tumour cells, not in metastatic tumour cells isolated from distal organs, thereby overlooking the difference in IL-33 expression metastatic versus primary tumour cells. Thus, while studying the IL-33 involvement in anti-cancer immunity, it’s important to specify the tumour type and clinical stage of the disease.

In summary, our data supports a new paradigm for cancer immunology where the IL-33/ILC2 axis acts as an effector pathway that assists and mediates anti-cancer immune responses and reduces tumour metastasis. IL-33 and/or ILC2s therefore may have important therapeutic utility amongst the constellation of cancer immunotherapies that are currently emerging.

### Experimental Procedures

#### Cell lines

All the cell lines were grown in Dulbecco’s modified Eagle medium (DMEM), supplemented with 10% heat-inactivated fetal bovine serum (FBS), 2 mM L-glutamine, 100 U/ml penicillin+100 μg/ml streptomycin (P+S), and 10 mM HEPES.

#### Murine lung tumour model

The primary TC1 tumour is a murine lung tumour model derived from primary lung epithelial cells of C57BL/6 mice immortalized using the amphotropic retrovirus vector LXSN16 carrying human papillomavirus genes E6/E7, and subsequently transformed with pVEJB plasmid expressing the activated human c-Ha-ras oncogene^22^. TC1 cells display high expression of TAP-1 and MHC-I. The metastatic A9 tumour was derived from the primary TC1 tumour cell line and display spontaneous down-regulation of MHC-I (or H2-K1) by immunoselection *in vivo* during immunization/challenge experiment^52^.

#### Murine prostate cells

The LMD cell line is an established cell line derived from metastatic mouse prostate cancer^53,54^, and it is TAP-1 and MHC-I deficient. The pTAP-1-EGFP-stably–transfected LMD cells^28^ are used to study the differential activation of TAP-1 promoter, which also indirectly correlates with MHC I expression on the LMD cell surface.

#### TC1 and A9 EGFP-expressing clones

A gene expression construct with full-length cDNA for the selected mouse IL-33 gene (NM_001164724.1) was produced using the pIRES2-EGFP vector (Clontech Lab). Primary TC1 and metastatic A9 cells were transfected with the pIRES2-EGFP-gene constructs using FuGene 6 transfection reagent (Promega) *in vitro*. Forty-eight hours after transfection, EGFP-positive cells were sorted by FACS (BD Aria II Cell Sorter) in order to obtain stable transfectants. Selection and expansion in culture was repeated twice before the cells were finally sorted into single-cell clones. Stably transfected clones were further isolated by FACS from a population of EGFP-positive cells.

#### Real-time (quantitative) polymerase chain reaction (qPCR)

Purified genomic DNA was used as a template for amplifications using 200 to 500 nM of each primer and 1 μL SYBR Green *Taq* ReadyMix (Roche) in a total 10 μL reaction mixture. Thirty-five to forty cycles of denaturation (5 s, 95 °C), annealing (5 s, 61 to 63 °C), and elongation (20 s, 72 °C) were carried out using a Roche LightCycler 480 instrument.

#### PCR arrays

Total cellular RNA was extracted from A9+vector, A9+IL-33, and TC1+vector murine lung carcinoma cell lines using Illustra RNAspin Mini Kit (GE Healthcare Life Science). Reverse transcription of 1 μg of total cellular RNA was performed using the reverse transcription kit (SSII RT) from Invitrogen. The cDNA synthesized was used to perform “The Mouse Cancer Inflammation & Immunity Crosstalk RT² Profiler PCR Array” (Qiagen, Cat # PAMM-181Z). Duplicate arrays were performed with preparations from A9+vector, A9+IL-33, and TC1+vector murine lung carcinoma cell lines. Relative changes in gene expression were calculated using the ΔΔCt (threshold cycle) method. Changes in cycle threshold (ΔCt) values for each tested gene were obtained by subtracting the average of the threshold cycle (Ct) values for the housekeeping genes (Gusb, Hsp90ab1, Gapdh, and Actβ) from the threshold cycle value of the gene. The expression fold change was calculated as (2^(−ΔΔCt)^). Expression of the housekeeping genes between the tumour cell samples (A9+vector; A9+IL-33; TC1+vector) were normalized to the transcription levels seen in the A9+vector samples. The ratio up- or down-regulation was then calculated relative to the normalized transcription of each gene in each of the tumour cell lines. Fold-change values greater than one indicate a positive- or an up-regulation. Fold-change values less than one indicate a negative or down-regulation.

#### Antibodies used for flow cytometry to check for chimerism in mice after bone marrow transplantation

FITC-conjugated CD45.1 (11-0453-81, #A-20) and PerCP-Cy5.5-conjugated CD45.2 (45-0454-80, #104) were purchased from Affymetrix eBioscience.

#### Antibody used to block Fc receptors

CD16/32 (564220, BD Pharmingen).

#### Excluding nonviable cells from flow cytometry

Fixable Viability Dye eFluor 780 (65-0865-14, Affymetrix eBioscience).

#### ILC2 isolation

The following antibodies were purchased from Affymetrix eBioscience: FITC-conjugated lineage marker monoclonal antibodies against: CD3 (Clone 17A2; 11-0032-82), CD8a (Clone 53-6.7; 11-0081-82), TCRb (Clone H57-597; 11-5961-82), CD19 (Clone eBio1D3; 11-0193-82), B220 (Clone RA3-6B2; 11-0452-82), NK1.1 (Clone PK136; 11-5941-82), Mac-1 (Clone M1/70; 11-0112-82), GR-1 (Clone RB6-8C5; 11-5931-82), and Ter119 (Clone TER-119; 115921-82); phycoerythrin (PE)-conjugated antibody against CD127 (Clone eBioSB/199; 12-1273-82); PerCP-Cy5.5-conjugated antibody against ST2 (Clone RMST2-2; 46-9335-82). BV605 AmCyan-conjugated antibody against Thy 1.2 (Clone 53-2.1; 140317) was purchased from BD Biosciences.

#### CD4, CD8 staining

APC-conjugated antibody against CD4 (553051, RM4-5, BD Bioscience) and PE-Cy7-conjugated antibody against CD8a (25-0081-82, 53-6.7, Affymetrix eBioscience).

#### H2-K1 expression

PE-conjugated anti-H2K1 (anti-K^b^) mouse monoclonal antibody (553570, BD Pharmingen).

#### Flow Cytometry

BD FACS Aria II was used for cell sorting and phenotypic analysis. The program FlowJo v.8.7 was used for data analysis.

Mice C57BL/6 (Cat# 000664), B6.Pep3b (B6.SJL-Ptprca Pepcb/BoyJ), RAG1^−/−^ (Cat #002216), OT-1 (C57BL/6- Tg(TcraTcrb)1100Mjb/J, Cat#003831)^55^ mice were purchased from the Jackson Laboratory. RORα^−/−^ (C57BL/6J-Rorasg-3J/J) mice are not available commercially, but were made by Fumio Takei and maintained at the British Columbia Cancer Research Centre (BCCRC). All animals were maintained in specific pathogen-free facilities: either the BCCRC animal facility or the Centre for Disease Modeling at the University of British Columbia (UBC). Mice were used between 4–8 weeks of age. All mouse experiments were approved by the Animal Care Committee at UBC. Animals were maintained and euthanized under humane conditions in accordance with the guidelines of the Canadian Council on Animal Care.

#### Bone marrow transplantation

B6.Pep3b mice were lethally irradiated (1,000 Rads) and then received transplantation of 1 × 10^7^ whole bone marrow cells from 4-week-old WT (C57BL/6 J) or RORα^−/−^ mice. Mice were given ciprofloxacin prophylactically for 4 weeks. The quality of the bone marrow transplantation was analysed by flow cytometry 8–16 weeks later by determining the ratio between CD45.1 and CD45.2 positive cells in peripheral blood. The efficiency of the bone marrow transplants was greater than 90% in all mice used (Supplementary Figure 1).

#### Splenocyte adoptive transfer

Spleen was placed into the 70 um cell strainer. Using the plunger end of the syringe, the spleen was mashed through the cell strainer. The cell strainer was washed with 5 mL DMEM. Cells were centrifuged for 5 min at 1500 rpm. Supernatant was removed and cell were re-suspended with ACK buffer (A10492-01, Gibco) and incubated at RT for 5 min. After neutralization with PBS, cells were washed once again. 0.81 × 10^6^ RORα^−/−^ splenocytes and 0.84 × 10^6^ WT splenocytes were injected IV into each RAG^−/−^ mouse. Four weeks later, 50ul of peripheral blood from each animal were collected to check for CD4+/CD8+ reconstitution, using flow cytometric analysis, before tumour establishment.

#### Tumour establishment

Genetically modified tumour cells were injected into WT or bone marrow chimeric animals subcutaneously (s.c.) into the right flank. Note for all experiments, 50 μl of 5 × 10^5^ tumour cells in HBSS (ThermoFisher Scientific) were used, with the exception of the adoptive transfer experiment where 50 µl of 5 × 10^4^ tumour cells were injected into recipients. Tumour growth was monitored by measuring tumour dimensions with calipers three times per week. Tumour volumes were calculated according to the equation tumour volume = length × width × height × π/6 with the length (mm) being the longer axis of the tumour. Animals were weighed at the time of tumour measurement. Mice were euthanized if they reached humane end point, based on 20% reduction in body weight, a tumour volume larger than 1 cm^3^ or ulceration of the tumour. At the humane end point, final animal weights and tumour volumes were calculated before mice were euthanized and tumours were resected and weighed. Tumours were monitored for up to the humane endpoint, which was around 21 days for A9 tumours and up to 38 days for TC1 tumours.

#### Primary Tissue Preparation

Cell suspensions were prepared from tumours, local lymph nodes (mesenteric, inguinal and lumbar) and lungs. Tissues were cut into small pieces with a razor and digested for 40 min in DMEM, 10% FBS, P+S, 50 mM 2-mercaptoethanol (2ME), Collagenase IV (Invitrogen), and DNase (Sigma) at 37 °C. Digested tissue was pushed through a 70 μm strainer, and Percoll (GE Healthcare) gradient enrichment of leukocytes followed.

#### Isolation and culture of ILC2 cells for analysis

ILC2s were isolated as previously described by Halim *et al*.^16^ and Halim *et al*.^26^. Single cells were incubated with 2.4G2 for blocking Fc receptors. After staining with FITC-conjugated lineage marker mAbs (CD3, CD8a, TCRb, CD19, B220, NK1.1, Mac-1, GR-1, and Ter119), PE-conjugated CD127, PerCP-Cy5.5-conjugated ST2, BV600 AmCyan-conjugated Thy 1.2 cells were purified/sorted by FACS. Purified ILC2s were then cultured in RPMI-1640 media containing 10% FBS, supplemented with penicillin/streptomycin and 50 mM 2-mercaptoethanol at 37 °C. Cells were stimulated with TSLP (10 ng/ml) and IL-33 (10 ng/ml) for 5–7 days prior to use.

#### Cytokine production assay

The secretion of IL-5, IL-13 was assessed by (eBioscience) enzyme-linked immunosorbent assays (ELISAs) according to the manufacturer’s protocol: IL-5 Ready-Set-go ELISA kit (88-7054-22, eBioscience); IL-13 Ready-Set-go ELISA kit (88-7137-88; eBioscience).

#### CTL assay and Granzyme b assay with ILC2s

Metastatic prostate tumour cells containing a green fluorescence protein (GFP) reporter gene under the TAP1 promoter (LMD+pTAP1/GFP) were used as the target cells for CTL-mediated killing. As previously shown, these cells do not express GFP in the absence of TAP1/MHC I expression^28^. 2 × 10^4^ LMD+pTAP1/GFP cells/well were cultured with and without 1 × 10^4^ activated ILC2s/well in a 96-well plate. ILC2s were isolated from lungs as previously described and activated with IL-33 and TSLP for 5–7 days prior to co-culturing with LMD cells. Cells were analyzed for the expression of EGFP by fluoresence microscopy (EVOS SL, Life Technology). After co-culture for 48 hours, freshly isolated CD8^+^DCs were added to wells (2 × 10^3^ cells/well), and the wells were pulsed for ~4 h with OVA (257–264) peptide at a concentration of 10ug/ml. CD8^+^ dendritic cells (DC) were isolated from C57Bl/6 mouse using the CD8^+^ DC Isolation kit for mouse (130-091-169, Miltenyi Biotec, Inc.). After the 4hr OVA treatment, freshly isolated CD8^+^ OT-1 T cells (4 × 10^4^ cells/well) were also added to the wells and the total cellular mixture was incubated together for four days. Splenic CD8^+^ T cells were isolated from OT-1 mouse using the Negative Selection EasySep CD8^+^ T cell Enrichment Kit (19753, StemCell Thechnologies). CD8^+^ T cells isolated from OT-1 mouse express a TCR that specifically recognizes OVA (257–264) (SIINFEKL) in the context of H2-K1.

After final incubation, the cells from each well were incubated with anti-mouse APC-eFluor 780-conjugated anti-mouse CD8a (47-0081-80, clone 53-6.7, Affymetrix eBioscience) and Granzyme B PE-conjugated (12-8898-80, Clone NGZB, Affymetrix eBioscience) using the Intracellular Fixation and Permeabilization Buffer Set (cat. 88-8824). Flow cytometry was used to assess the expression of Granzyme b in CTLs.

#### Immunohistochemistry

Resected tumours were embedded in Tissue-Tek O.C.T. media (Sakura) on dry ice and immediately stored at −80 °C until sectioning. Ten micron (10 µm) thick sections were collected on Leica cryostat and stored at −80 °C until staining. Slides were removed from −80 °C, fixed in cold acetone or acetone:methanol. Following washing in Tris-buffered saline (TBS, pH 7.4), slides were incubated with protein block and subsequently incubated with specific antibodies overnight. Appropriate horseradish peroxidase (HRP) conjugated secondary antibodies were used for detection of the primaries and developed with DAB chromogen. Slides were counter stained with haematoxylin and eosin (H&E) and dehydrated in ethanol and xylene. Giemsa staining was used to detect eosinophils. Slides were then cover-slipped and imaged with an Aperio ScanScope at 20x magnification. Tumours were embedded in Tissue-Tek O.C.T. media on dry ice and immediately stored at −80 °C until sectioning. 5 µm thick sections were collected on a Leica cryostat and stored at −80 °C until staining. Slides were removed from −80 °C, fixed in cold acetone. Following washing in TBS, slides were incubated with protein block and subsequently incubated with specific antibodies overnight. Antibodies used: anti-CD4 (553043; BD Bioscience), anti-CD8 (553027; BD Bioscience), anti-FoxP3 (54501; Abcam), anti- Ly-6G (MAB1037; R&D System), anti-CD68 (53444; Abcam). Appropriate horseradish peroxidase (HRP) conjugated secondary antibodies were used for detection of the primary antibodies. The slides were then developed with either DAB or StayRed chromogen. Slides were counter-stained with haematoxylin and dehydrated in ethanol and xylene. Slides were then cover-slipped and imaged with an Aperio ScanScope at 20x magnification.

#### Statistics

Data were analyzed with Excel. A Student’s t test was used for determining statistical significance between groups; p ≤ 0.05 was considered significant. Error bars were calculated as standard error of the mean.

#### Animal Ethics

All animals were maintained in specific pathogen-free facilities: either the British Columbia Cancer Research Centre (BCCRC) animal facility or the Centre for Disease Modeling at the University of British Columbia (UBC). All mouse experiments were approved by the Animal Care Committee and veterinarians at UBC, who oversee both animal facilities. Animals were maintained and euthanized under humane conditions in accordance with the guidelines of the Canadian Council on Animal Care (CCAC).

## Electronic supplementary material

Supplemental Figure 1
