# Supplementary material for: Type 2 Innate Lymphocytes Actuate Immunity Against Tumours and Limit Cancer Metastasis
Source: Sci Rep. 2018 Feb 13;8:2924. doi: 10.1038/s41598-018-20608-6 (PMC5811448; doi:10.1038/s41598-018-20608-6)
Supplement: Supplementary file 1 — Supplemental Figure 1 [file 41598_2018_20608_MOESM1_ESM.pdf]

NAME OF JOURNAL: Scientific Reports

MANUSCRIPT NUMBER: SREP-17-31215A

TITLE OF MANUSCRIPT: Type 2 Innate Lymphocytes Actuate Immunity Against Tumours and Limit Cancer Metastasis

AUTHORS: Iryna Saranchova, Jeffrey Han, Rysa Zaman, Hitesh Arora, Hui Huang, Franz Fenninger, Kyung Bok Choi, Lonna Munro, Cheryl G. Pfeifer, Ian Welch, Fumio Takei, Wilfred A. Jefferies*

CORRESPONDING AUTHOR'S NAME: Wilfred A. Jefferies

Supplementary Figure:

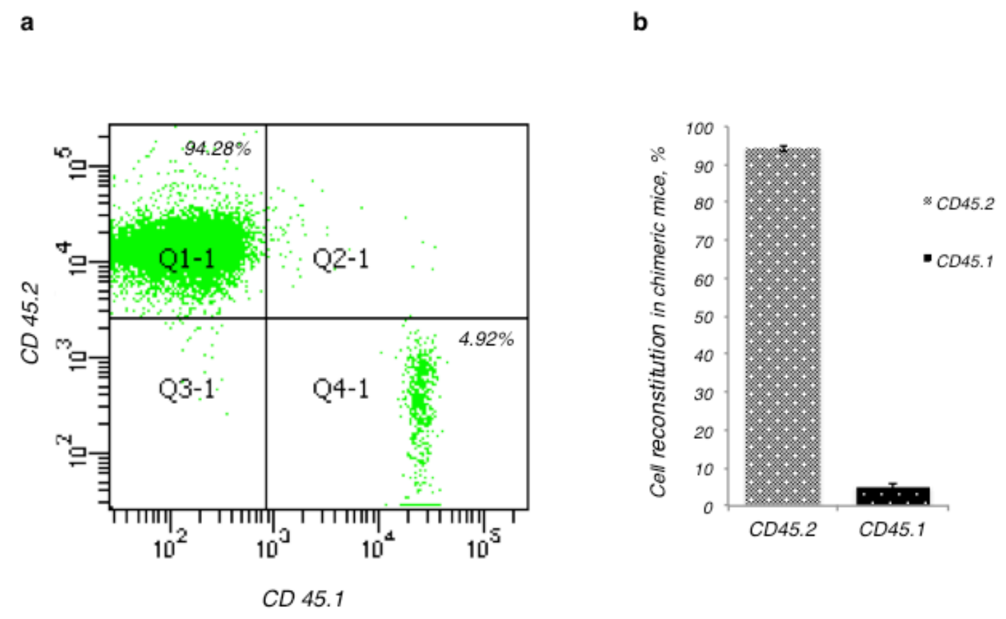

Supplemental Figure 1: Generation of bone marrow chimeras. Bone marrow chimeras were generated by reconstitution of lethally irradiated B6.Pep3b (CD45.1) mice with whole bone marrow cells from either wild type or RORα$^{-/-}$ (both CD45.2) mice. (a) Quality of the bone marrow transplantation was analyzed by flow cytometry determining the ratio between CD45.1 and CD45.2 positive cells in peripheral blood. (b) All the transplants were between 92-96% efficient.
